# Determination of the polarization states of an arbitrary polarized terahertz beam: Vectorial vortex analysis

**DOI:** 10.1038/srep09416

**Published:** 2015-03-24

**Authors:** Toshitaka Wakayama, Takeshi Higashiguchi, Hiroki Oikawa, Kazuyuki Sakaue, Masakazu Washio, Motoki Yonemura, Toru Yoshizawa, J. Scott Tyo, Yukitoshi Otani

**Affiliations:** 1 School of Biomedical Engineering, Saitama Medical University, Yamane 1397-1, Hidaka, Saitama 350-1241, Japan; 2 Department of Advanced Interdisciplinary Sciences, Utsunomiya University, Yoto 7-1-2, Utsunomiya, Tochigi 321-8585, Japan; 3 Center for Optical Research & Education (CORE), Utsunomiya University, Yoto 7-1-2, Utsunomiya, Tochigi 321-8585, Japan; 4 Research Institute for Science and Engineering, Waseda University, Kikuicho 17, Shinjuku, Tokyo 162-0044, Japan; 5 NPO 3D Associates, Kishiya 4-13-18, Yokohama, Japan; 6 College of Optical Sciences, The University of Arizona, Tucson, AZ 85721, USA

## Abstract

Vectorial vortex analysis is used to determine the polarization states of an arbitrarily polarized terahertz (0.1–1.6 THz) beam using THz achromatic axially symmetric wave (TAS) plates, which have a phase retardance of Δ = 163° and are made of polytetrafluorethylene. Polarized THz beams are converted into THz vectorial vortex beams with no spatial or wavelength dispersion, and the unknown polarization states of the incident THz beams are reconstructed. The polarization determination is also demonstrated at frequencies of 0.16 and 0.36 THz. The results obtained by solving the inverse source problem agree with the values used in the experiments. This vectorial vortex analysis enables a determination of the polarization states of the incident THz beam from the THz image. The polarization states of the beams are estimated after they pass through the TAS plates. The results validate this new approach to polarization detection for intense THz sources. It could find application in such cutting edge areas of physics as nonlinear THz photonics and plasmon excitation, because TAS plates not only instantaneously elucidate the polarization of an enclosed THz beam but can also passively control THz vectorial vortex beams.

The polarization states of a beam can be varied by the optical anisotropy of materials using the electro- and magnetic-optical effects as external triggers. In addition, optical scattering and reflection processes can create changes in polarization. Furthermore, variations in vibrational and rotational spectral structure can be induced by light-matter interactions, due to the external electrical field of a polarized laser beam. For these reasons, polarization determination is important in materials science^1^, pharmacology^2^, and even astronomy^3^. The frequency response of a polarized beam permits elucidation of atomic and molecular dynamics^4^, stereo and chiral structures of a protein^5^, and charge density distributions^6^. Interestingly, the use of THz-wave polarization pulse shaping with arbitrary vector control to examine molecular dynamics has been reported in the last few years^7^,^8^. The vector control of electric fields has been realized, enabling the use of nonlinear optical crystals and femtosecond lasers. Terahertz time-domain spectroscopy (THz-TDS) enables sequential electrical fields to be obtained with a high time resolution.

Vectorial vortex (VV) beam technology is able to control a beam's spatially variable polarization and phase, and can generate longitudinal electrical fields^9^, optical vortices^10^,^11^,^12^,^13^, and higher order Poincaré beams^14^ carrying orbital angular momentum. This technology has led to exciting discoveries and applications such as electron acceleration^9^,^15^, optical trapping^16^, super-resolution microscopy^17^,^18^, and even the creation of new functional materials including metamaterials^19^ with photo-alignment^20^,^21^ and chiral nanostructures^22^. The information describing the light-matter interactions is contained in the VV beam. By decoding this information, it should be possible to understand the light-matter interaction in more detail. Intense THz beams generated using gyrotrons, free electron lasers (FELs), and coherent synchrotron radiation (CSR) in particular have attracted interest for their use as next-generation beams in cutting-edge areas of physics such as nuclear fusion, isotope separation, and electron acceleration. To examine light-matter interactions using intense THz beams, it is important to determine the polarization characteristics^23^,^24^,^25^. However, conventional methods cannot easily obtain the polarization states of a beam from an intense THz source, because it is necessary to modulate the polarization both mechanically and electrically^26^. Even THz-TDS is not suitable for characterizing the electrical fields from such sources. Instead it is necessary to instantaneously elucidate the polarization states of intense THz sources.

In this paper, VV analysis is used to determine the polarization states of arbitrarily polarized 0.1–1.6 THz beams using achromatic axially symmetric wave plates, which have a phase retardance of Δ = 163° and are made of polytetrafluorethylene (PTFE). Linearly polarized beams are converted into THz vectorial vortex (TVV) beams having no spatial and wavelength dispersion. The polarization states are reconstructed for incident THz beams of unknown but uniform polarization. The polarization determination is also demonstrated at frequencies of 0.16 and 0.36 THz. Furthermore, the polarization states of TVV beams are estimated after they pass through THz achromatic axially-symmetric wave (TAS) plates. The results obtained by solving the inverse source problem agree with the values used in experiments.

## Results

### Determination of the polarization states of an arbitrarily polarized THz beam

**Figure 1:**
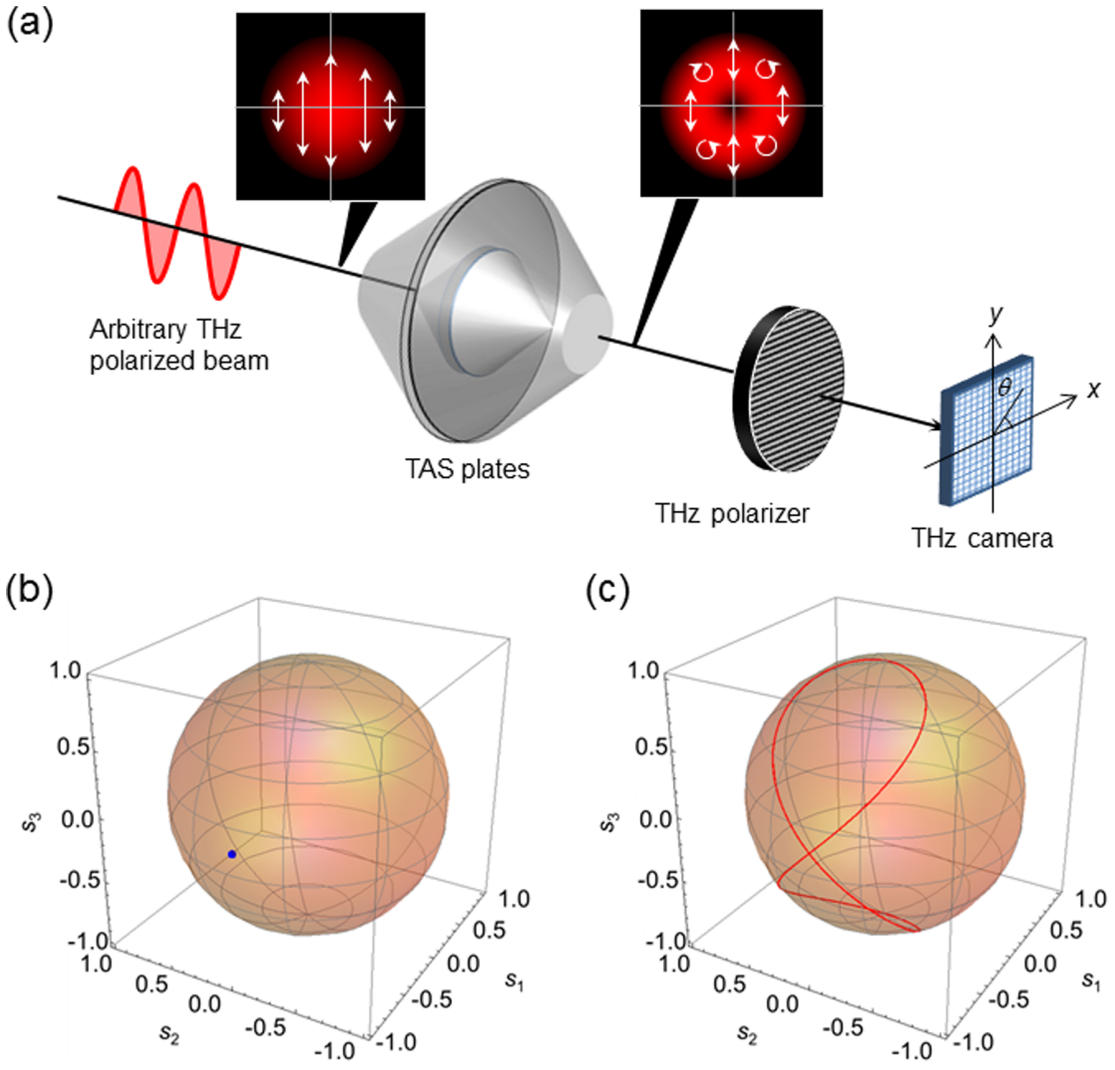
(a) Polarization determination for an arbitrarily polarized beam. When a THz beam is incident on the TAS plates and THz polarizer, the THz camera captures the distributions of the spatial beam intensity. The linearly polarized beam is subsequently converted into a VV beam. Panels (b) and (c) indicate the polarization states on the Poincaré sphere normalized by *s*_0_. In panel (b) starting at (−1,0,0), the polarization state becomes a figure of eight on the Poincaré sphere in panel (c).

As shown in Fig. 1(a), TAS plates, a THz polarizer, and a THz camera are used to determine the polarization state of an arbitrary but uniformly polarized THz beam. Consider an incident THz beam linearly polarized at 90°, represented by a dot on the Poincaré sphere in Fig. 1(b). After passing through a pair of TAS plates with an integrated retardance of 90°, the polarization states are axisymmetrically modulated by a function of the azimuthal angle *θ* of the beam. The spatially varying polarizations are represented as a figure of eight on the Poincaré sphere shown in Fig. 1(c). The output passes through a linear polarizer oriented at angle *θ*_0._ The resulting Stokes vector ***S***_1_(*r*, *θ*) is given by ***S***_1_(*r*, *θ*) = ***P***(*θ*_0_)·***T***(*θ*)·***S***_0_, where *S*_0_ = (*s*_00_, *s*_01_, *s*_02_, *s*_03_)*^T^* is the Stokes vector of an incident THz beam of unknown polarization state, ***T****(θ)* is the Mueller matrix for the TAS plates, *r* is the radial direction, ***P***(*θ*_0_) is the Mueller matrix for the polarizer^27^,^28^, and 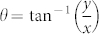.

The output beam is analyzed by a linear polarizer oriented at *θ*_0_ = 0°. The resulting intensity distribution *I*(*r*, *θ*) varies periodically with a frequency of 2*θ* and 4*θ*, i.e., 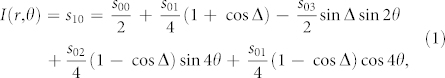
where, *s*_10_ indicates the *s*_0_ component of the Stokes parameter after passing through the polarizer, *Δ* is the retardance of the TAS plates, and 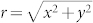. The data are fitted using a Fourier series 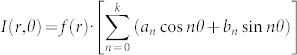, where *f*(*r*) is a constant of proportionality and the Fourier coefficients *a*_0_, *b*_2_, *a*_4_, and *b*_4_ are evaluated to estimate the Stokes parameters *s*_00_, *s*_01_, *s*_02_, and *s*_03_ for the incident THz beam. The ellipticity and azimuth are determined from the Stokes parameters in Ref. 28. Using this method, the Stokes parameters for the TVV beams can be measured from a THz image. The standard method for analyzing the polarization state of an arbitrary beam is to rotate a retarder in front of a linear polarization analyzer and take several intensity measurements. The TAS plates allow this modulation to occur angularly, and the polarization properties of the beam can be estimated from a single image, that is, a snapshot. The TAS plates effectively provide both the basis of the polarization determination and the TVV beam control. Proof-of-principle experiments are conducted as follows.

**Figure 2:**
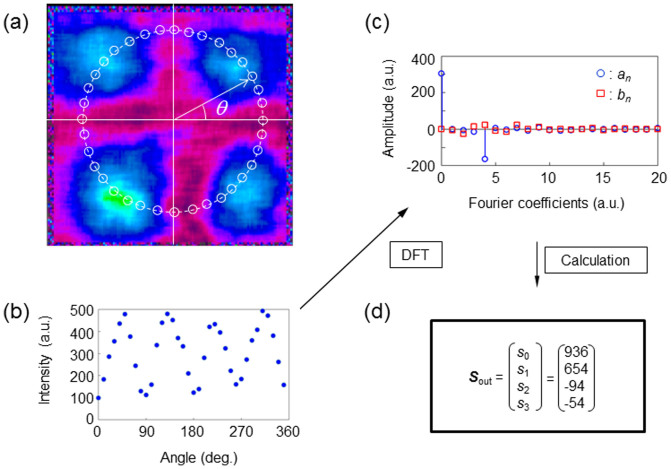
Analytical procedure for determining the polarization by VV beam control. (a) Image of a THz beam of unknown polarization. The intensity distribution of the THz beam is measured by the THz camera after the beam passes through the THz polarizer. The distribution is modulated in angle *θ*. Using this image, the intensity distribution at each angle *θ* is plotted in panel (b). (c) Amplitude spectra following a discrete Fourier transform (DFT). (d) From the amplitude spectra, the Stokes parameters are evaluated.

Figure 2(a) illustrates the conversion of a linearly polarized beam into a TVV beam. An experiment is performed to demonstrate that the system can be used in reverse to determine the polarization of a uniformly polarized THz beam. A linearly polarized THz beam passes through the TAS plates as shown in Fig. 1. The polarization states of the arbitrarily polarized THz beams are modulated by the plates. The intensity distribution is captured by a pyroelectric camera and plotted in Fig. 2(b) after extracting values from the 2D image. The intensity varies periodically with a frequency 2*θ* and 4*θ*. After calculating the discrete Fourier transform, the Fourier coefficients *a_n_* and *b_n_* are evaluated as shown in Fig. 2(c). Figure 2(d) lists the Stokes parameters for the THz beam evaluated from the captured image.

**Figure 3:**
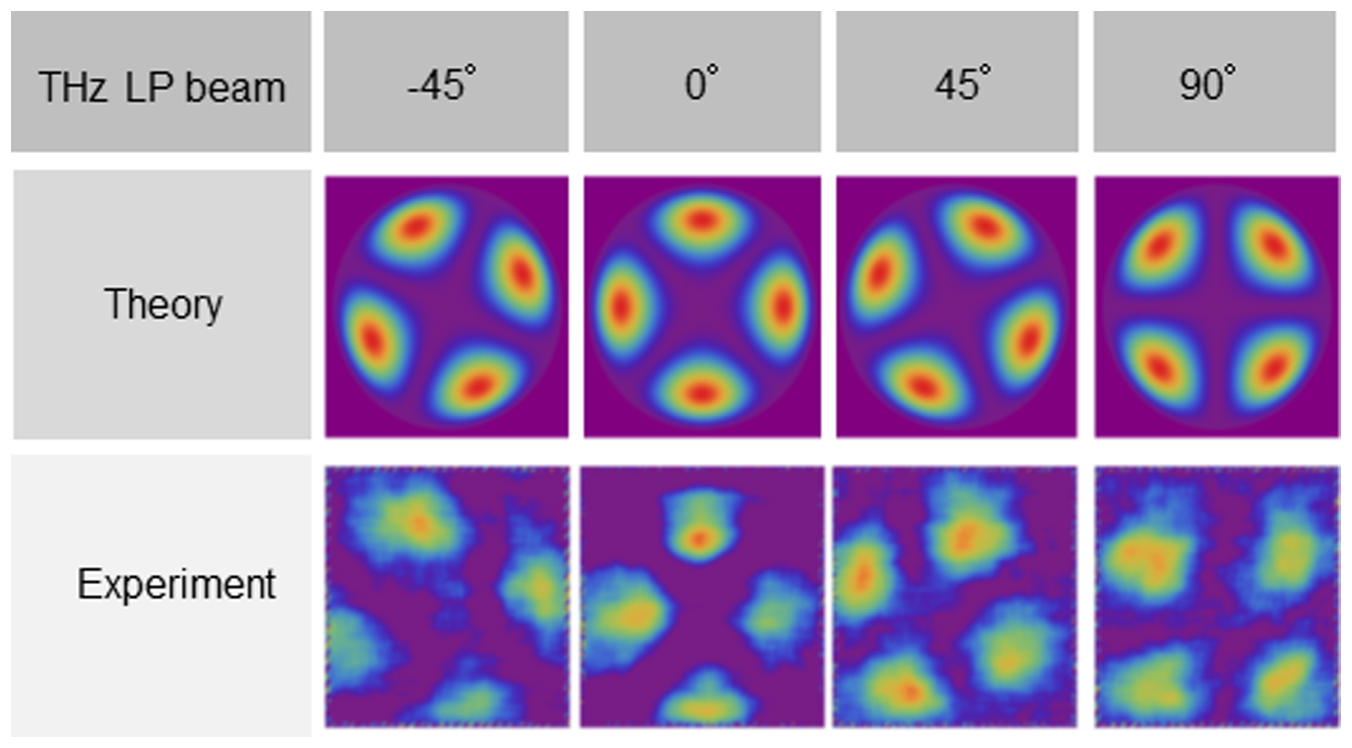
Comparison between theoretical values and experimental results for THz beams with typical polarizations of −45°, 0°, 45°, and 90°.

**Figure 4:**
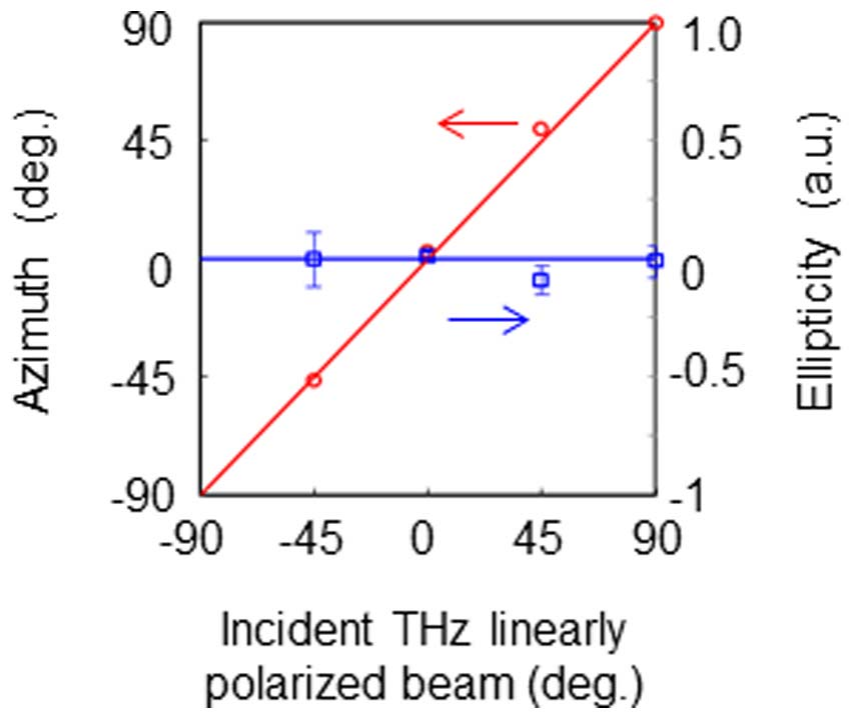
Measurements (symbols) and theoretical results (solid lines) of the ellipticity (in blue) and of the azimuth (in red) for the data in Fig. 3. The ellipticities of the incident THz beams are almost zero. The azimuths of the incident THz beams are −46.3°, 2.2°, 49.3°, and 89.7°. The standard deviations of the ellipticities and their azimuths are 0.06 and 0.25°, respectively. These values validate the agreement between the experimental and theoretical results.

Uniform arbitrary polarization states of the incident THz beams are also analyzed. A mechanical rotation stage changes the polarization of the beams by rotating the THz source. The incident beams are polarized at typical values of −45°, 0°, 45°, and 90°. Figure 3 shows theoretical results compared to measured THz images. The THz beams are converted into TVV beams and modulated according to the incident polarization states. The experimental images agree with the theoretical results. Using the polarization analysis shown in Fig. 2, the Stokes parameters, the degree of polarization (DOP), the ellipticity, and its azimuth are analyzed. The theoretical values and experimental results are given in Table 1, and the ellipticities and azimuths are plotted in Fig. 4. The ellipticities of the incident beams are almost zero. The azimuths of the incident THz beams are found to be −46.3°, 2.2°, 49.3°, and 89.7°, respectively. The standard deviations of the ellipticities and azimuths are 0.06 and 0.25°, respectively. Note that *s*_02_/*s*_00_ = 0.06 ± 0.00 in the third line of Table 1 because *s*_02_/*s*_00_ = 0.06 ± 0.004(6) and the *s*_00_ components in Table 1 are *s*_00_(−45°) = 711, *s*_00_(0°) = 1121, *s*_00_(45°) = 780, and *s*_00_(90°) = 1086.

**Figure 5:**
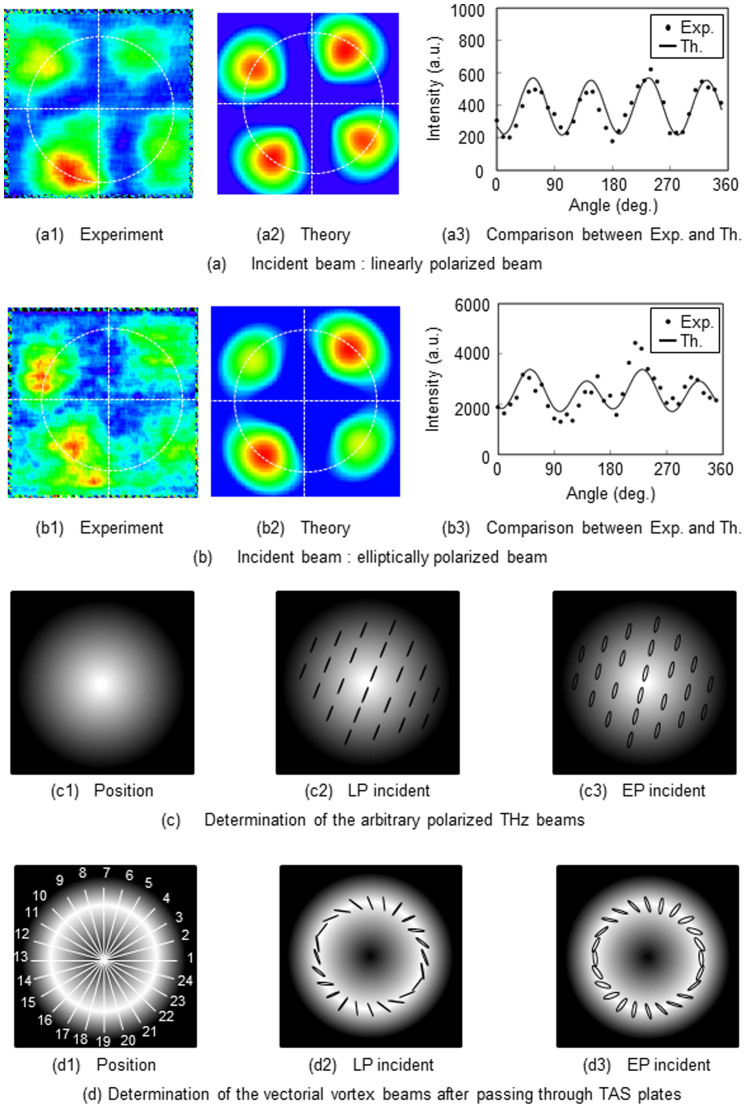
Polarized beam determination as an inverse source problem. (a) Results for a linearly polarized incident beam. The Stokes parameters are (790, −248, 248, 54)*^T^* using the data in panel (a1). (a2) Reconstructed intensity distributions from these Stokes parameters. (a3) Comparison between the intensity profiles obtained from the intensity values on the circles in panels (a1) and (a2). (b) Results for an elliptically polarized incident beam. The polarization state is again determined from the THz image in panel (b1). (b2) Intensity distribution reconstructed from the Stokes parameters (4953, −1283, 632, 1376)*^T^*. (b3) Comparison between experiment and theory. (c) Determination of arbitrary incident polarized THz beams. (c1) Intensity distribution for an incident beam having a Gaussian profile. (c2) Linearly polarized beam with *ε* = 0.05 and *ϕ* = 67.5°. (c3) Elliptically polarized beam with *ε* = 0.22 and *ϕ* = 76.9°. The emerging beam has been divided into the 24 angular wedges shown in panel (d1). (d2) and (d3) Transmitted polarization states of the vectorial vortex beam after passing through the TAS plates for incident beams of linear and elliptical polarization. The linear and elliptical shapes represent the polarization states of the beams.

A proof-of-principle experiment is conducted for the inverse source problem. An elliptically polarized beam is used for this purpose, because it is not easy to generate circularly polarized THz beams. The experimental results are presented in Fig. 5. Firstly, compare linearly and elliptically polarized incident beams on the TAS plates. When the incident beam is linearly polarized, the THz image is captured by the camera after passing through the TAS plates and the THz polarizer, as shown in Fig. 5(a1). Stokes parameters of (790, −248, 248, 54)*^T^* are computed from the image. By substituting these parameters into Eq. (1), the intensity distributions are reconstructed theoretically in Fig. 5(a2). The experimental and theoretical intensity distributions about angle *θ* are determined from the intensity values on the circles respectively shown in Figs. 5(a1) and 5(a2). The experimental and theoretical distributions plotted in Fig. 5(a3) agree with each other. Next, elliptical incident polarization is used to confirm the theory for arbitrarily polarized THz beams. The incident beam is elliptically polarized using a Fresnel rhomb made of high-density polyethylene (HDPE) positioned before the TAS plate. The polarization state is then determined by the TAS plates as shown in Fig. 5(b1). Calculated Stokes parameters of (4953, −1283, 632, 1376)*^T^* are found for the incident beam. As shown in Fig. 5(b2), the intensity distributions are again reconstructed, as in the linear polarization case. A comparison between experiment and theory is graphed in Fig. 5(b3). The experimental and theoretical results are in good agreement. Details of the incident polarization states are sketched in Fig. 5(c). Figure 5(c1) shows the intensity distribution of an incident beam having a Gaussian profile and arbitrary polarization state. Using the preceding method, the polarization states of the incident THz beams can be determined, as shown in Figs. 5(c2) and 5(c3). Figure 5(c2) indicates a linearly polarized beam with ellipticity *ε* = 0.05 and azimuth *ϕ* = 67.5°. Figure 5(c3) shows an elliptically polarized beam with ellipticity *ε* = 0.22 and azimuth *ϕ* = 76.9°. Since the method can determine the polarization states without use of electrical or mechanical components, the polarization sates of the vectorial vortex beam can also be determined using 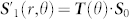 after it passes through the TAS plates. The polarization states of an incident arbitrarily polarized beam are known from the THz images with angular control. The transmitted beam is divided into 24 angular wedges as shown in Fig. 5(d1). The Stokes parameters and ellipsometric parameters are calculated at those angles. Figures 5(d2) and 5(d3) show the output polarization states of the vectorial vortex beam after passing through the TAS plates for linear and elliptical polarizations, respectively. The linear and elliptical shapes in Figs. 5(c2), 5(c3), 5(d2), and 5(d3) represent the polarization states of the beams. It is thus possible to determine not only the polarization states of the incident beam, but also the polarization states of the VV beam after passing through the TAS plates.

**Figure 6:**
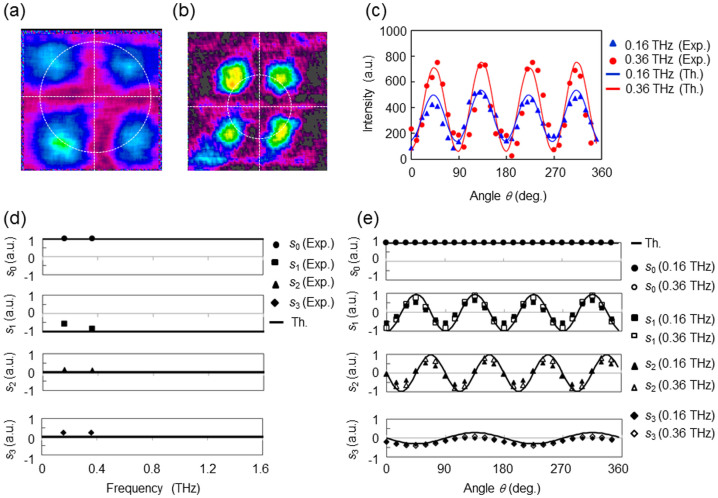
Demonstrations of polarization determination at frequencies of 0.16 and 0.36 THz. (a) and (b) Intensity distributions captured by the THz camera. (c) Comparison between experiment and theory. (d) Stokes parameters obtained at 0.16 and 0.36 THz with *s*_0.16 THz_ = (664, −380, 78, 128)*^T^* and *s*_0.36 THz_ = (812, −688, 78, 148)*^T^*. The Stokes parameters are normalized by *s*_0_. (e) Polarization states of the vectorial vortex beam transmitted through the TAS plates.

Consider the frequency dependence of the polarization determination in the THz region. The expected spectral range is 0.1–1.6 THz because the refractive index of PTFE is constant at 1.44, although the power absorption of PTFE is less than 0.8 according to Ref. 32. An experiment to determine arbitrary polarization states is conducted at different THz frequencies. Two THz sources at frequencies of 0.16 and 0.36 THz are used for this purpose, with linearly polarized beams at 90°. Figures 6(a) and 6(b) illustrate the intensity distribution captured by the THz camera. The polarization states of an incident polarized beam are independently determined from the two intensity distributions. The two intensity profiles along angle *θ* in Fig. 6(c) are similar in shape, despite their different amplitudes and biases. (The output powers of the two THz sources are different.) The resulting Stokes parameters are graphed in Fig. 6(d). The Stokes vectors are determined to be *s*_0.16 THz_ = (664, −380, 78, 128)*^T^* and *s*_0.36 THz_ = (812, −688, 78, 148)*^T^*. However, the ellipticity and azimuth are nearly the same, *ε*_0.16 THz_ = 0.12, *ϕ*_0.16 THz_ = 84.2° and *ε*_0.36 THz_ = 0.10, *ϕ*_0.36 THz_ = 86.8°. Therefore, PTFE is a suitable THz material for determining the polarization states of an incident THz beam in the 0.1 to 1.6 THz region. After passing through the TAS plates, the parameters become those plotted in Fig. 6(e). The symbols and curves respectively indicate the experimental results and numerically simulated values. (The Stokes parameters are normalized by their *s*_00_ components.) According to Fig. 6(e), the experimental results are in good agreement with the theoretical results at 0.16 and 0.36 THz.

## Discussion

Polarization determination can solve the inverse source problem associated with the conversion from arbitrary polarized THz beams to TVV beams. The simulations indicate that a phase retardance of *Δ* = 163° by fourth-order Fresnel reflections occurs at a slope angle of *β* = 55° for TAS plates made of PTFE. The ellipticity and the azimuth distributions for a TVV beam can be determined using the rotating polarizer method. Arbitrarily polarized THz beams can be analyzed. The measured values agree with theory, although imperfections in the optical components lead to some depolarization that is not accounted for in the inversion algorithms.

The method can determine not only the arbitrary polarization state of an incident beam, but also the polarization state of a VV beam after transmitting through the TAS plates. Polarization can be determined using PTFE across the spectral range from 0.1 to 1.6 THz. It is important, however, to account for the absorption coefficient of the THz materials. The accuracy of the determination of the Stokes parameters is estimated to be about ±0.1. The precision and resolution of the measured parameters could be improved by stabilizing the THz source and camera, and by refining the THz alignment.

It is possible to extend the investigations to beams produced by intense THz sources such as gyrotrons, FELs, and CSR. The present method enables not only the examination of light-matter interactions through polarization determination, but also the clarification of additional polarization properties of intense THz beams such as TVV beam control. That could find application to nonlinear plasmon excitation, isotope separation, and electron acceleration. The method also suggests a new universal concept for polarization detection for all wavelength regions from UV to THz.

The deterioration of the DOP is influenced by the extinction in the THz polarizer, by the sensitivity of the pyroelectric camera, and by the accuracy of the THz alignment. Also, crosstalk of the polarization occurs when the TVV beam is focused into the pyroelectric camera, as well as polarization scrambling (depolarization) by the thickness of the PTFE at the center of the TAS plates, which is comparable to the wavelength of the THz beams at *λ* = 1.875 mm. The theoretical analysis assumes that the entire system is composed of ideal retarders and polarizers. In practice, depolarizing effects should be accounted for in the system calibration to improve the accuracy of the polarization estimates. If the fabrication and performance of the TAS plates and other elements could be improved, the Mueller matrices of the system components would more closely match those for an ideal setup and the overall system accuracy would improve. A similar effect has been noted for infrared polarimeters using polarizers with low extinction ratios^29^. In spite of these issues, the method convincingly demonstrates TVV beam control, and the components can be used as the basis of an angularly modulated polarization detector. With proper calibration, even partially polarized THz beams can be measured.

## Method

### THz achromatic axially symmetric wave plate (TAS plate)

Based on the Fresnel reflection coefficients, the total internal reflection of a THz beam introduces a phase retardance *δ*(*n*,*β*) between the *p* and *s* orthogonal polarization states given by
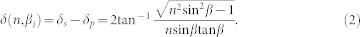
Here *n* and *β* are the refractive index of the material and the incident angle, respectively^30^. Whereas *β* is a constant, *n* depends on the wavelength *λ*. The output beam is totally internally reflected and produces a phase retardance of *δ* (*n*,*β*) between the *p* and *s* states in the THz region^27^,^30^.

**Figure 7:**
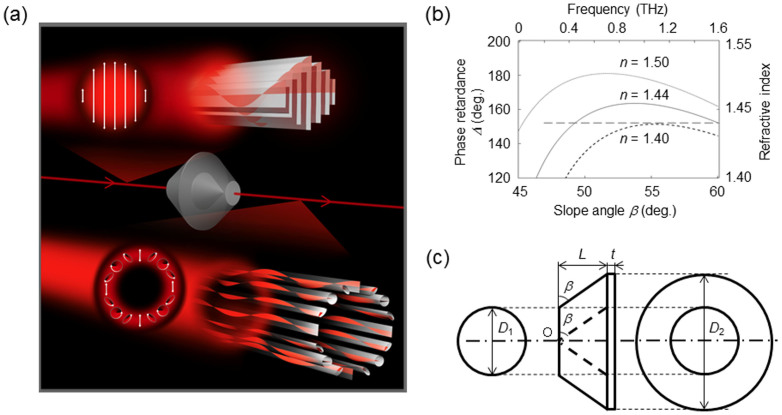
Achromatic axially symmetric wave plates for the 0.1–1.6 THz region. (a) Illustration of TVV control. The linearly polarized beam is converted into a VV beam after passing through the TAS plates. (b) Simulated results for internal Fresnel reflections in the THz region. The long-dashed line marks the refractive index, whose value is almost the same in the THz region as in Ref. 32. Calculations compare the phase retardance generated by four reflections as a function of the slope angle for three different refractive indices (dashed curve: *n* = 1.40, solid curve: *n* = 1.44, and dotted curve: *n* = 1.50). (c) Trihedral figure of the TAS plates.

Figure 7 sketches the experiments. When an arbitrarily polarized THz beam is incident on a TAS plate with a concave conical surface (similar to an element rotated about the optical axis), the reflected beam is transformed into a conical beam after reflecting off the sloping surface. The beam then becomes ring-shaped, because the reflection is omnidirectionally generated along the optical axis shown in Fig. 7(a). The polarization states of the beam are axially symmetrically modulated as a function of the azimuthal angle *θ* of the beam, converting it into a VV beam.

### Rotating analyzer method

The relationship between the input *S*_2_ and output *S*_3_ Stokes vectors after the VV conversion is
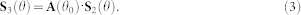
Through the action of the angularly varying Mueller matrix in Eq. (3), the polarization state of the TVV can be passively controlled. The Stokes parameters are determined to be
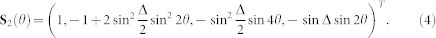
The spatially variable intensity distribution in a 2D image is 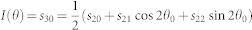, where, *s*_30_ is the *s*_0_ component of the Stokes parameters of the output THz beam. Fourier analysis determines the Stokes parameters *S*_20_, *S*_21_ and *S*_22_ from the angularly varying data measured from an image. These parameters are calculated and the resulting ellipticity and its azimuth are 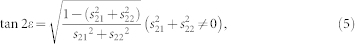
and 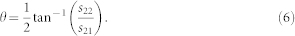


### Experimental setup

In Fig. 1, the experimental setup performs two functions: it generates the TVV beams and it analyzes the polarization state of an arbitrarily polarized THz beam. The system consists of a THz source with a frequency *ν* = 0.16 THz, a linear polarizer (from CDP), the spectrum of which is measured by a THz Michelson interferometer, two lenses (*f* = 100 mm made of PTFE, model LAT100 from Thorlabs), a pair of TAS plates, a rotating wired grid polarizer (model MWG20-II from Joint Technology Development Platform), and a pyroelectric camera (model PYIII-C-B from Ophir-Spiricon). To generate the TVV beams, the THz polarized beam collimated by the planoconvex lens is directed onto the TAS plates. Four azimuthally varying total internal reflections inside the TAS plates convert the incident polarized beam into a TVV beam. A double-convex lens, a rotating polarizer with transmittance angle *θ*_0_, and a pyroelectric camera are used to evaluate the 2D distribution of polarization states of the beam. Alternatively, the TAS plates, double-convex lens, static polarizer, and camera constitute an angularly modulated polarimeter to estimate the unknown polarization state of the incident beam. If a different value for the retardance is chosen, the size of the figure of eight on the Poincaré spheres shown in Figs. 1(b) and 1(c) changes. For example, when the total phase retardance is *Δ* = 90°, radial and azimuthal polarized beams are generated, as described in Ref. 31. Uniformly polarized beams can also be converted into various types of TVV beams by controlling the internal reflections.

### Simulation of phase retardance in the THz region

To manipulate the phase retardance using internal Fresnel reflections, the necessary slope angles *β* for a TAS plate are calculated using the material properties of PTFE. The long-dashed line in Fig. 7(b) indicates the refractive index *n* = 1.44 in the 0.1–1.6 THz region^32^. Three phase retardances are determined by the refractive indices of *n* = 1.40, 1.44, and 1.55. The relationship between the slope angle and the phase retardance is graphed as the three curves in Fig. 7(b). Assuming the refractive index to be *n* = 1.44, the slope angle is estimated assuming a small angle dependence. The phase retardance is *δ* = 40.75° due to a single reflection in the case of *β* = 55°, which implies the total phase retardance is *Δ* = 163.0° after four reflections. Figure 7(c) is a schematic of a TAS plate which has dimensions of *D*_1_ = 50 mm, *D*_2_ = 100 mm, *L* = 35.7 mm, and *t* = 5 mm. The plate was manufactured on a precision lathe.

### Evaluation of TVV beams by the rotating analyzer method

**Figure 8:**
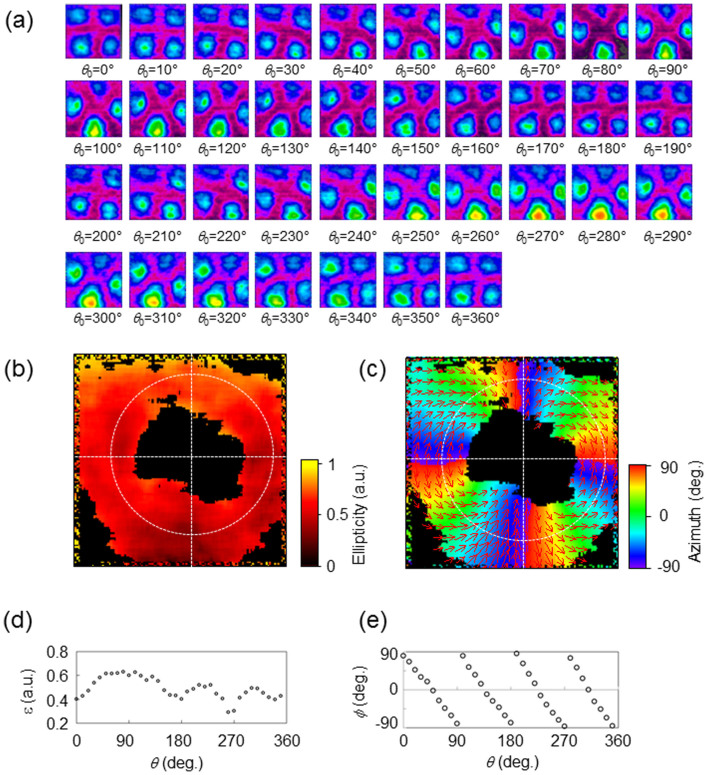
(a) Images of the VV beams obtained using the rotating polarizer method. The intensity distributions vary smoothly with the angle of rotation of the polarizer. (b) Distribution of the absolute value of the ellipticity. (c) Distribution of the azimuthal angle. (d) and (e) Variation of the ellipticity and its azimuth along the angle *θ* using their values from panels (b) and (c).

To demonstrate that a TAS plate can be used for the passive control of TVV beams, the rotating polarizer method is employed. Figure 8(a) depicts the intensity distributions of the output beam as the angle *θ*_0_ is varied by rotating the polarizer. The distribution rotates smoothly with the angle. The ellipticity and azimuth distributions are shown in Figs. 8(b) and 8(c). Figures 8(d) and 8(e) plot the angular variation in the ellipticity and azimuth, using the data in Figs. 8(b) and 8(c). The ellipticity has a frequency of 4*θ*. The change in the azimuth follows the change in angle *θ*. The change in the ellipticity with the angle *θ* has a bias component of 0.4. Since the polarization states of the VV beam are spatially scrambled, the extinction ratio cannot be eliminated, as observed at the center of the VV beams in Fig. 8(a).

### Check for the inverse source problem

**Figure 9:**
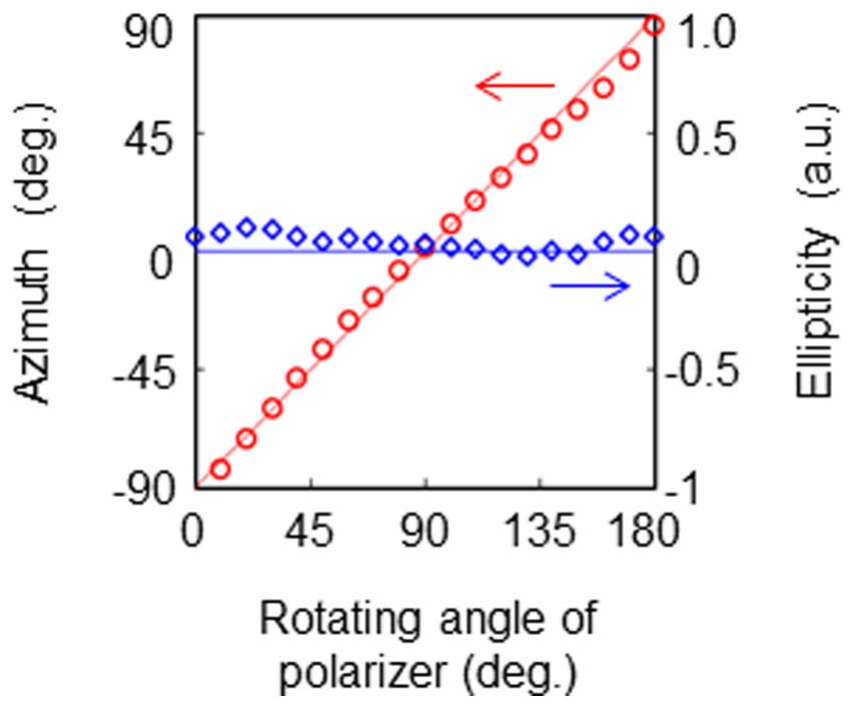
Check for the inverse source solution using linear system theory. To determine the inverse problem, the ellipsometric parameters are recalculated from the THz images captured by rotating the THz polarizer through the coordinate transformation of the system shown in Fig. 1(a). The theoretical parameters are plotted as the red line for the azimuth and the blue line for the ellipticity. The experimental values are given by the red circles for the azimuth and by the blue diamonds for the ellipticity. The ellipticities are almost zero, whereas the azimuth varies linearly.

To determine the inverse source solution using linear system theory, the THz images are recalculated by rotating the THz polarizer through the coordinate transformation of the system sketched in Fig. 1(a). Figure 9 graphs both the measured results and the theoretical values of the ellipticity and its azimuth. The ellipticity is nearly zero, while the azimuth varies linearly. The experimental results agree with the theory, demonstrating the proof-of-principle for polarization determination using the inverse source conversion from a linearly polarized beam to a VV beam.

## Author Contributions

T.W., T.H. and Y.O. conceived the algorithm. T.W., T.H., H.O. and K.S. designed and built the system. T.W., T.H. and H.O. performed the experiments and analyzed the data. T.W., T.H., M.W., M.Y., T.Y., J.S.T. and Y.O. conceived the experiments and discussed the results.

## Figures and Tables

**Table 1:** Comparison between experimental and theoretical values for arbitrary polarized THz beams

Incident LP		*s*_0_/*s*_0_	*s*_1_/*s*_0_	*s*_2_/*s*_0_	*s*_3_/*s*_0_	DOP	Ellipticity	Azimuth (deg.)
−45°	Exp.	1	−0.03 ± 0.02	−0.66 ± 0.08	0.00 ± 0.19	0.66 ± 0.07	0.00 ± 0.11	−46.3 ± 0.9
	(Th.)	(1)	(0)	(−1)	(0)	(1)	(0)	(−45)
0°	Exp.	1	0.83 ± 0.04	0.06 ± 0.00	0.01 ± 0.04	0.83 ± 0.04	0.01 ± 0.02	2.2 ± 0.2
	(Th.)	(1)	(1)	(0)	(0)	(1)	(0)	(0)
45°	Exp.	1	−0.17 ± 0.02	1.14 ± 0.02	−0.20 ± 0.13	1.17 ± 0.04	−0.09 ± 0.06	49.3 ± 0.5
	(Th.)	(1)	(0)	(1)	(0)	(1)	(0)	(45)
90°	Exp.	1	−1.15 ± 0.03	0.01 ± 0.01	−0.03 ± 0.15	1.15 ± 0.03	−0.01 ± 0.07	89.7 ± 0.3
	(Th.)	(1)	(−1)	(0)	(0)	(1)	(0)	(90)
